# A proteomics informed by transcriptomics insight into the proteome of *Ornithodoros erraticus* adult tick saliva

**DOI:** 10.1186/s13071-021-05118-1

**Published:** 2022-01-03

**Authors:** Ricardo Pérez-Sánchez, Angel Carnero-Morán, M. Luz Valero, Ana Oleaga

**Affiliations:** 1 Parasitology Laboratory, Spanish National Research Council Institute of Natural Resources and Agrobiology (CSIC-IRNASA), Cordel de Merinas, 40-52, 37008 Salamanca, Spain; 2 Proteomics Section, Central Service for Experimental Research, University of Valencia, Carrer del Dr. Moliner, 50, 46100 Burjassot, Spain

**Keywords:** *Ornithodoros erraticus*, Soft ticks, Saliva, Proteome, Proteomics, LC–MS/MS, SWATH-MS

## Abstract

**Background:**

The argasid tick *Ornithodoros erraticus* is the main vector of tick-borne human relapsing fever (TBRF) and African swine fever (ASF) in the Mediterranean Basin. The prevention and control of these diseases would greatly benefit from the elimination of *O. erraticus* populations, and anti-tick vaccines are envisaged as an effective and sustainable alternative to chemical acaricide usage for tick control. *Ornithodoros erraticus* saliva contains bioactive proteins that play essential functions in tick feeding and host defence modulation, which may contribute to host infection by tick-borne pathogens. Hence, these proteins could be candidate antigen targets for the development of vaccines aimed at the control and prevention of *O. erraticus* infestations and the diseases this tick transmits. The objective of the present work was to obtain and characterise the proteome of the saliva of *O. erraticus* adult ticks as a means to identify and select novel salivary antigen targets.

**Methods:**

A proteomics informed by transcriptomics (PIT) approach was applied to analyse samples of female and male saliva separately using the previously obtained *O. erraticus* sialotranscriptome as a reference database and two different mass spectrometry techniques, namely liquid chromatography–tandem mass spectrometry (LC–MS/MS) in data-dependent acquisition mode and sequential window acquisition of all theoretical fragment ion spectra MS (SWATH-MS).

**Results:**

Up to 264 and 263 proteins were identified by LC–MS/MS in the saliva of *O. erraticus* female and male ticks, respectively, totalling 387 non-redundant proteins. Of these, 224 were further quantified by SWATH-MS in the saliva of both male and female ticks. Quantified proteins were classified into 23 functional categories and their abundance compared between sexes. Heme/iron-binding proteins, protease inhibitors, proteases, lipocalins and immune-related proteins were the categories most abundantly expressed in females, while glycolytic enzymes, protease inhibitors and lipocalins were the most abundantly expressed in males. Ninety-seven proteins were differentially expressed between the sexes, of which 37 and 60 were overexpressed in females and males, respectively.

**Conclusions:**

The PIT approach demonstrated its usefulness for proteomics studies of *O. erraticus*, a non-model organism without genomic sequences available, allowing the publication of the first comprehensive proteome of the saliva of *O. erraticus* reported to date. These findings confirm important quantitative differences between sexes in the *O. erraticus* saliva proteome, unveil novel salivary proteins and functions at the tick–host feeding interface and improve our understanding of the physiology of feeding in *O. erraticus* ticks. The integration of *O. erraticus* sialoproteomic and sialotranscriptomic data will drive a more rational selection of salivary candidates as antigen targets for the development of vaccines aimed at the control of *O. erraticus* infestations and the diseases it transmits.

**Graphical Abstract:**

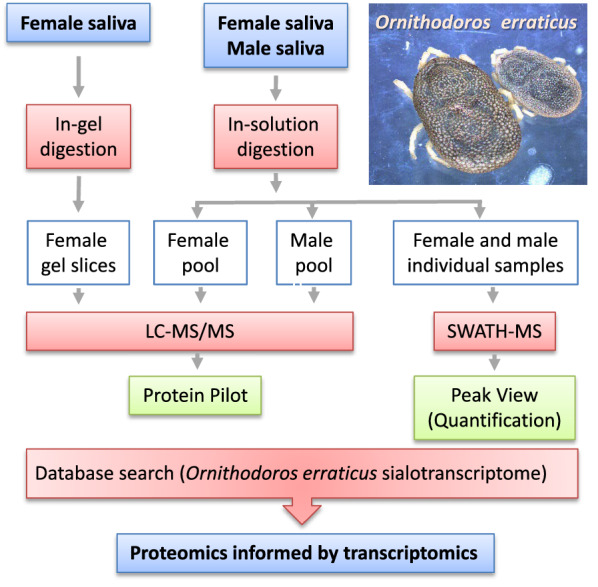

**Supplementary Information:**

The online version contains supplementary material available at 10.1186/s13071-021-05118-1.

## Background

Ticks are an increasing medical and veterinary concern because they efficiently transmit a large range of infectious agents, including viruses, bacteria and protozoa, which cause serious diseases to humans and domestic and wild animals and result in important economic losses worldwide [1, 2].

The argasid tick *Ornithodoros erraticus* has been reported in the Iberian Peninsula, northern and western Africa and western Asia [3]. In the Mediterranean Basin, this tick is the main vector of the African swine fever (ASF) virus and of several *Borrelia* spp. spirochetes that cause tick-borne human relapsing fever (TBRF) [4, 5]. In this region, *O. erraticus* colonises anthropic environments and lives in close association with swine on free-range pig farms, hidden inside and around pig premises, which facilitates the transmission and persistence of ASF and TBRF in affected areas [3, 6, 7]. Along its biological cycle, *O. erraticus* passes through the stages of eggs, larvae, nymphs and adults. Similar to most argasid ticks, it is a fast feeder, taking between 10 and 30 min to complete its blood meal, and can ingest up to five- to tenfold its unfed body weight in blood. Adults feed independently: they do not co-feed and there is no evidence that male assists female to feed. After feeding and mating, females lay a batch of approximately 120 eggs, and they can repeat this trophogonic cycle up to 10 times [3, 6, 7]. *Ornithodoros erraticus* has three to five nymphal stages (N1–5) depending on the environment, with each moult requiring a blood meal. The adult males can result from moults from N3 to N5, but females emerge from N4 to N5. The life-cycle can be completed in as little as 5 months under optimal conditions, but takes between 2 and 5 years in the field. Additionally, adults can survive for at least 5 years without feeding. This fasting tolerance increases the ability of the species to act as a pathogen reservoir [3, 6, 7]. *Ornithodoros erraticus* is also the type-species of the “*Ornithodoros erraticus* complex”, and some species belonging to this complex, such as *O. asperus*, *O. lahorensis*, *O. tartakovsky* and *O. tholozani*, are distributed through the Middle East, the Caucasus, the Russian Federation and the Far East, where they transmit local species of TBRF-causing borreliae [1, 8, 9]. In the last decade, the ASF virus has spread across this region in an uncontrolled manner, from the Caucasus to China, where there is a suspicion that local tick species belonging to the *O. erraticus* complex might also be competent vectors for the ASF virus [10–14]. If this theory is confirmed, the presence of these ticks in anthropic environments could significantly contribute to the transmission and persistence of ASF throughout this vast area, greatly complicating its prevention and control. Thus, any potentially effective strategy aimed at controlling ASF and TBRF will require the elimination of *Ornithodoros* vector populations from at least the anthropic environments.

Anti-tick vaccines have proven to be an effective and sustainable method for the control of tick infestations and tick-borne diseases with clear advantages over the application of chemical acaricides [15–18]. Moreover, the efficacy of acaricide application against *Ornithodoros* ticks is seriously limited due to their endophilic/nidicolous lifestyle, which makes these ticks less accessible to the effects of chemical acaricides [19].

Success in tick vaccine development is largely dependent on the identification of new and highly protective tick antigens. The search for new candidate protective antigens is currently being directed towards tick molecules that play important biological functions at the tick–host interface and, more precisely among the salivary and intestinal proteins involved in biological processes that have been specifically evolved by ticks to adapt to haematophagy [20–26].

Accordingly, next-generation sequencing (NGS) and high-throughput proteomics technologies are being used to explore the transcriptome and proteome of the salivary glands/saliva and midguts of an increasing number of tick species, thus obtaining the corresponding sialomes and mialomes [24–29]. These studies have identified a wealth of tick molecules related to tick haematophagy, tick–host interplay and pathogen transmission, which can then be scrutinised and filtered in vaccinomics pipelines for the selection of candidate protective antigens [27, 30–34].

In *O. erraticus*, the recent application of omics technologies has allowed us to obtain the transcriptome and proteome of the female tick midgut before and after blood-feeding [35, 36]. The resulting integrated mialome has been examined following a reverse vaccinology approach, and several vaccine candidate antigens have been identified, produced in recombinant form and tested for protection efficacy in animal immunisation trials. Some of these candidates have proven to be protective against *Ornithodoros* spp. vectors and are therefore potentially useful for inclusion in vaccine formulations for tick control [22, 23].

Similarly, in order to characterise the *O. erraticus* sialome, we have recently obtained the salivary transcriptome of female ticks and analysed the salivary gene expression dynamics throughout the female trophogonic cycle [26]. The resulting sialotranscriptome is the first comprehensive set of protein-coding messenger RNA (mRNA) sequences expressed in *O. erraticus* salivary glands, and has shown high complexity and functional redundancy, similar to that observed in the sialotranscriptomes of other soft and hard ticks [28, 37]. Given that *O. erraticus* is a non-model organism and its genome has not been sequenced, this annotated sialotranscriptome constitutes an invaluable reference database for any proteomics study of the salivary glands and/or saliva of *O. erraticus*. The use of tissue-specific databases derived from RNA-sequencing data as a reference database for large-scale protein identification is called proteomics informed by transcriptomics (PIT) [38], and this approach is being increasingly applied in studies involving tick saliva following the expansion of tick sialotranscriptomic analyses using NGS methods [39–41].

As *O. erraticus* saliva must contain all of the bioactive molecules required by the tick to successfully feed, decoding its composition will lead to the discovery of new antigen targets for the development of vaccines for the control and prevention of *O. erraticus* infestations and the diseases it transmits. Therefore, the objective of the present work was to obtain the proteome of the saliva of *O. erraticus* adult ticks. To this end, we have used a PIT approach to analyse female and male saliva separately using two different mass spectrometry approaches: liquid chromatography–tandem mass spectrometry (LC–MS/MS) in data-dependent acquisition (DDA) mode, and sequential window acquisition of all theoretical fragment ion spectra mass spectrometry (SWATH-MS). SWATH-MS is a specific variant of data-independent acquisition (DIA) methods which combines deep proteome coverage capabilities with quantitative consistency and accuracy [42].

Herein, we report the identification of 387 non-redundant proteins in the saliva of *O. erraticus* adult ticks and perform a qualitative and quantitative comparison of the saliva protein composition between both sexes, including a brief discussion on the protein functional groups and families that are more abundantly expressed in each sex. The integration of *O. erraticus* sialoproteomic and sialotranscriptomic datasets facilitates a better understanding of the physiology of feeding in *O. erraticus* and will drive the discovery of new and more effective antigen targets for the development of anti-tick vaccines.

## Methods

### Ticks

The *O. erraticus* ticks were obtained from the laboratory colony of CSIC-IRNASA (Salamanca, Spain), which was initiated from specimens captured in nature in Salamanca Province (Spain) in the 1980s. The colony was maintained under conditions of 28 °C, 85% relative humidity (RH) and 12-h light/12-h dark photoperiod and fed regularly on New Zealand white rabbits. All protocols involving tick feeding and rabbit handling were approved by the Ethical and Animal Welfare Committee of the CSIC-IRNASA and met the corresponding EU legislation (Directive 2010/63/EU; http://data.europa.eu/eli/dir/2010/63/oj).

### Saliva collection

Saliva samples were collected separately from newly moulted 3-month-old female and male ticks after saliva secretion had been stimulated with 1% pilocarpine, following the protocol described by Diaz-Martín et al. [43], with minor modifications. Batches of 20–30 ticks were first washed by successive immersions in tap water, 3% hydrogen peroxide, two washes in distilled water, 70% ethanol and two more washes in distilled water, following which they were dried on paper towels and kept at 28 °C and 85% RH. The ticks were then immobilised ventral-side up on a glass plate using double-sided adhesive tape in groups of five individuals. Female ticks were administered 1 µl of 1% pilocarpine hydrochloride (Sigma Chemical Co., St. Louis, MO, USA) in phosphate-buffered saline (PBS) pH 7.4 through the genital pore; male ticks were administered 0.5 µl of 1% pilocarpine hydrochloride through the anal pore. For injections, a 5-µl-volume Hamilton syringe and 33-gauge, 25-mm-long needle were used. Soon after stimulation, ticks started to move the chelicerae and emit small droplets of clear viscous saliva. To collect saliva droplets, 1 μl of PBS was placed on the tick mouthparts using a micropipette, and immediately harvested and deposited on 50 μl of ice-cooled PBS. Saliva was continuously collected until perceptible emission stopped, usually 15–20 min after stimulation. Three replicated biological saliva samples from each sex were prepared, each containing the secretion of 20 female ticks per sample (F1, F2, F3) and 30 male ticks per sample (M1, M2, M3). Saliva samples were centrifuged for 20 min at 12,000 *g* and 4 °C, and the supernatants were recovered and stored at − 20 °C. Protein concentration was measured as a function of absorbance at 280 nm in the NanoDrop 2000 spectrophotometer (Thermo Fisher Scientific, Waltham, MA, USA), and sample reproducibility within and between sexes was checked by sodium dodecyl sulphate-polyacrylamide gel electrophoresis (SDS-PAGE) in silver-stained 5–20% gradient gels following standard methods.

### Protein digestion and sample preparation

Trypsin digestion and proteomic analyses of saliva samples were carried out at the Central Service of Support to Experimental Research (SCSIE) of the University of Valencia Proteomics Unit, a member of the ISCIII ProteoRed Proteomics Platform.

Salivary proteins were first digested in-solution with Sequencing Grade Trypsin (Promega, Madison, WI, USA) as follows. First, a 10-μg aliquot of each saliva sample (F1, F2, F3, M1, M2, M3) was reduced by incubation with 10 mM dithiothreitol (DTT) (Sigma Chemical Co.) in a total final volume of 100 µl of 50 mM ammonium bicarbonate (ABC) (Sigma Chemical Co.) for 20 min at 60°C. The proteins were alkylated with 5.5 mM iodoacetamide (IAM) (Sigma Chemical Corp.) in a final volume of 110 µl of 50 mM ABC for 30 min at room temperature in the dark. An excess of IAM was quenched by adding 100 µl of 20 mM DTT in 50 mM ABC and incubating the solution for 1 h at 37 °C. Each sample was then treated with 400 ng of trypsin in a final volume of 218 µl and incubated overnight at 37 °C. The digestion was stopped with 20 µl of 10% trifluoroacetic acid (TFA) (Thermo Fisher Scientific) in water. The mixtures were dried in a rotatory evaporator and dissolved in a final volume of 40 µl of 50 mM ABC.

A pool of female saliva samples (F1 + F2 + F3) was subjected to in-gel digestion following the protocol of Shevchenko et al. [44]. Briefly, 20 μg of pooled female saliva was resolved by 5–20% gradient SDS-PAGE and stained with Coomassie Blue. The lanes were sliced into three pieces according to the observed protein band pattern, with lane 1 containing 300- to 100-kDa bands; lane 2, 100- to 25-kDa bands; and lane 3, 25- to 10-kDa bands. Gel slices were reduced with DTT, alkylated with IAM and digested with 20 ng/μl of trypsin overnight at 37 °C. Digestion was stopped with 10% TFA at a final concentration of 0.1%, and the supernatants were filtered through a 0.22-μm filter and dried by vacuum centrifugation. Pellets containing the digested peptides were re-suspended in 11 μl (slices 1 and 3) or 6 μl (slice 2) of 2% acetonitrile, 0.1% TFA.

### LC–MS/MS analysis

The peptides recovered from trypsin digestions were separated by liquid chromatography (LC) using a NanoLC 425 LC systems (Eksigent Technologies LLC, Dublin, CA, USA) and analysed in a microESI qQTOF mass spectrometer (TripleTOF 6600+ system; AB Sciex LLC, Redwood City, CA, USA) connected in direct injection mode.

For the LC–MS/MS analysis of female and male saliva samples digested in-solution, two pools of samples were made, with one containing a 2-µl aliquot from each digested female sample (F1, F2, F3) and the other containing a 2-µl aliquot from each digested male sample (M1, M2, M3). A 5-μl sample of each pool was loaded onto a trap column (3 µm; C18-CL; 120 Å; 350 µm × 0.5 mm; Eksigent Technologies LLC) and desalted with 0.1% TFA at 5 µl/min for 5 min. The peptides were then loaded onto an analytical LC column (3 µm; C18-CL; 120 Ᾰ; 0.075 × 150 mm; Eksigent Technologies), equilibrated in 5% acetonitrile (ACN) (Thermo Fisher Scientific) and 0.1% formic acid (FA) (Thermo Fisher Scientific) and eluted using a linear gradient of 7–40% of buffer B (0.1% FA in ACN) in buffer A (0.1% FA in water) for 45 min at a flow rate of 300 nl/min. The eluted peptides were ionised in a OptiFlow Turbo Ion Source system (Sciex) (< 1 µl Nano) with 3.0 kV applied to the spray emitter. The triple time-of-fligh (TOF) analyser was operated in a DDA mode. Survey MS1 scans were acquired in the mass range of *m*/*z* 350–1400 for 250 ms in positive ion mode; the top 100 most intense ions were selected for fragmentation, and MS2 scans were acquired in the mass range of m/z 100–1500 for 25 ms in “high sensitivity” mode. Ions of charge 2+ to 4+ with a minimum intensity of 100 counts per second were selected for fragmentation. The rolling collision energy equations were set for all ions as for 2+ ions.

For the analysis of pooled female saliva digested in-gel, 5 µl of each digested gel slice (S1, S2, S3) was analysed as described above in a 20-min LC–MS/MS run.

### SWATH-MS acquisition

The acquisition of SWATH-MS data from female and male saliva samples was accomplished with the same microESI qQTOF mass spectrometer used for LC–MS/MS (TripleTOF 6600+ system; AB Sciex LLC) operated in swath mode. The six samples (F1, F2, F3, M1, M2, M3) were loaded in a random order to avoid bias in the analysis. A 5-μl aliquot of each digested sample was individually loaded onto a trap column (LC column; 12 nm; 3 µm; Triart-C18; 0.5 × 5.0 mm; YMC Co. Ltd., Kyoto, Japan) and desalted with 0.1% TFA at 10 µl/min for 5 min. The peptides were then loaded onto an analytical column (LC column; Luna Omega; 3 µm; Polar C18; 150 × 0.3 mm; Capillary; Phenomenex, Torrance, CA, USA) equilibrated in 3% ACN, 0.1% FA, and eluted with a linear gradient of 3–35% buffer B (0.1% FA in ACN) in buffer A (0.1% FA in water) for 45 min at a flow rate of 5 μl/min. Samples were ionised in a OptiFlow Turbo Ion Source system (1–50 µl micro), with 4.5 kV applied to the spray emitter; the analysis was carried out in DIA mode. Survey MS1 scans were acquired from 400 to 1250 *m*/*z* for 250 ms, followed by 25-ms product ion scans from 100 to 1500 *m*/*z* in ‘high sensitivity’ mode throughout 100 overlapping windows covering from 400 to 1250 *m*/*z*. The quadrupole resolution was set to ‘UNIT’ for the MS2 experiments, and the total cycle time was 2.79 s.

### Protein identification and quantification

After LC–MS/MS, the SCIEX.wiff data files were processed using the ProteinPilot v5.0 search engine (AB Sciex LLC). The three SCIEX.wiff files resulting from the LC–MS/MS runs of the three gel slices containing the pooled female saliva digested in-gel were combined in a single search.

The Paragon algorithm [45] of ProteinPilot was used to search the protein Fasta database derived from the recently published *O. erraticus* sialotranscriptome [26], which included 102,625 predicted proteins, of which 22,007 were high-confidence predictions and 18,959 were annotated (BioProject ID: PRJNA666995). Searches were done with trypsin specificity and IAM cys-alkylation, and the search effort was set to rapid.

The identified proteins were grouped based on MS/MS spectra by the ProteinPilot Pro Group™ Algorithm, regardless of the peptide sequence assigned, which avoided using the same spectral data in more than one protein. The protein within each group that could explain more spectral data with confidence was taken as the primary protein of the group.

Among the proteins identified by LC–MS/MS in DDA mode in the saliva samples, only those showing a ProteinPilot unused score > 1.3 (≥ 95% confidence) and a false discovery rate (FDR) < 1% were considered significant and included in subsequent analyses. Then, protein hits to non-annotated predicted proteins in the sialotranscriptome database were removed, and redundant identifications were manually removed by selecting the protein hit with the highest score as representative.

The resulting ProteinPilot group file contained all of the female and male spectral data, and this file was used as the reference spectral library to quantify proteins from the SWATH-MS raw data using PeakView 2.2 (AB Sciex LLC). Peptide areas were extracted from SWATH-MS runs for peptides with a confidence threshold > 95% and FDR < 1%. The retention times were aligned among the different samples using the main protein peptides. In addition, six transitions per peptide and a maximum of 20 peptides per protein were automatically selected for quantitation. Protein areas were calculated as the sum of the corresponding peptide areas. The quantitative data obtained by PeakView (protein areas) were normalised by total area using MarkerView (v1.3; AB Sciex LLC) and used for differential expression analysis.

The proteins quantified by SWATH-MS were filtered in a way similar to that for the proteins identified by LC–MS/MS in DDA mode; namely hits to non-annotated proteins and redundant identifications were removed.

The MS proteomics data have been deposited to the ProteomeXchange Consortium via the PRIDE partner repository [46] with the dataset identifier PXD027367.

### Functional annotation and classification of the identified proteins

Functional annotation of the proteins identified was performed using the UniProt KB database. The UniProt IDs of the proteins were used to extract the Gene Ontology (GO) terms for biological processes, molecular functions and cellular components, as well as cross-references in the InterPro, Pfam and Panther databases. The identified proteins were then functionally classified according to GO terms and bibliographic information, taking the classification applied by Kim et al. [41] in their study of the proteome of *Amblyomma americanum* tick saliva as a model.

### Differential expression and statistical analysis

The normalised protein areas were analysed by MultiExperiment Viewer (MeV) (http://www.tm4.org/mev.html) to identify the proteins that are differentially expressed in saliva between female and male ticks using Welch’s t-test subjected to Bonferroni correction. Salivary proteins showing an adjusted *P* value < 0.05 were considered significantly differentially expressed between female and male ticks. The mean quantity of every protein in each sex was calculated as the mean signal peak area in the three replicated saliva samples from each sex, and the fold-change in expression between female and male saliva was expressed as the ratio between the mean signal peak area in female and males. Hierarchical clustering analysis was performed using MeV, and the results of this analysis of the differentially expressed proteins of female and male samples were shown using a heat map after* Z*-score normalisation using Euclidean distances.

**Fig. 1:**
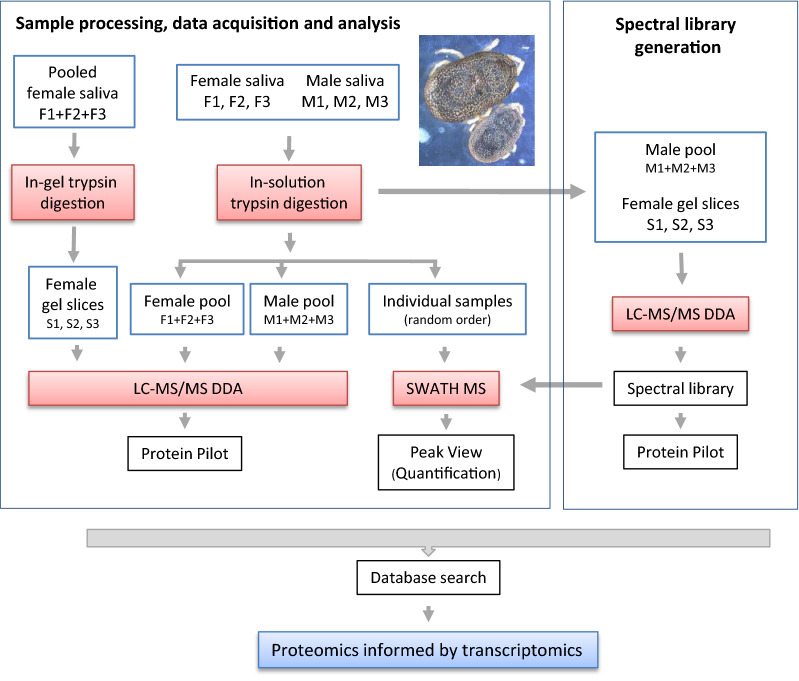
Schematics of experimental workflow employed in this study

The workflow for sample processing, data acquisition and analysis and spectral library generation is schematically represented in Fig. 1.

## Results and discussion

### SDS-PAGE of saliva from female and male *O. erraticus* ticks

Our aim in this study was to obtain and compare, both qualitatively and quantitatively, the proteome of the saliva of *O. erraticus* female and male ticks. To this end, we prepared three replicated saliva samples from each sex (F1, F2, F3 from females and M1, M2, M3 from males), which were first examined by SDS-PAGE to check reproducibility and then subjected to LC–MS/MS and SWATH-MS analyses.

**Fig. 2:**
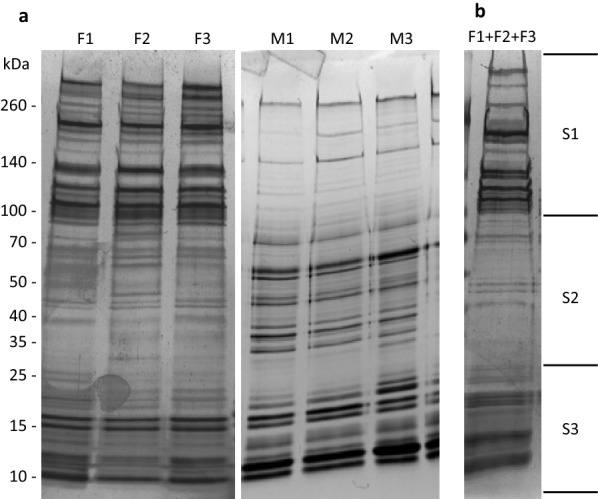
**a** Silver-stained 5–20% SDS-PAGE gel showing replicated samples of saliva (4 µg/lane) from female (F1, F2, F3) and male (M1, M2, M3) *Ornithodoros erraticus* ticks. **b** Coomassie Blue-stained 5–20% SDS-PAGE gel showing pooled female saliva (F1 + F2 + F3). The gel lane was sliced into the three pieces, and the resulting gel slices (S1, S2, S3) were digested with trypsin and analysed by LC–MS/MS

SDS-PAGE of saliva samples showed protein band patterns that were very similar within each sex and noticeably different between the sexes (Fig. 2). Both sexes displayed numerous bands with molecular weights ranging from > 260 to 10 kDa. However, in female saliva, the more numerous and intense bands were in the range of 300 to 100 kDa, while the more numerous and intense bands in male saliva ranged from 100 to 10 kDa. These band patterns indicate good reproducibility among samples of the same sex and anticipate some differences in the saliva protein composition between the sexes.

### Proteins identified by DDA LC–MS/MS

Female and male saliva samples digested in-solution were analysed by LC–MS/MS in DDA mode in two separate pools. Up to 469 protein hits were detected in the male saliva pool (Additional file 1: Table S1), but only 332 protein hits were detected in the equivalent female saliva pool (data not shown). This difference might be related to the different protein band patterns observed in the SDS-PAGE gel between female and male saliva (Fig. 2). In females, proteins larger than 100 kDa seemed somewhat more abundant than the rest and could have masked the identification of the less abundant proteins (Fig. 2a). Thus, to achieve a better characterisation of the female saliva proteome, pooled female saliva samples were resolved by SDS-PAGE, fractionated into three slices (Fig. 2b) and each slice analysed by LC–MS/MS in DDA mode. This analysis yielded 470 female protein hits (Additional file 1: Table S1). Because of the better results, we used the information obtained from the in-gel analysis for female protein annotation in the spectral library. ProteinPilot reports showing the spectrum, peptide and protein data in *O. erraticus* female and male ticks were generated and can be accessed in Additional file 2: Dataset S1 and Additional file 3: Dataset S2, respectively.

The information obtained from in-solution processed male saliva and from in-gel processed female saliva was combined to generate the reference spectral library for analysis of the SWATH-MS acquired data, which can be accessed in Additional file 4: Dataset S3.

**Fig. 3:**
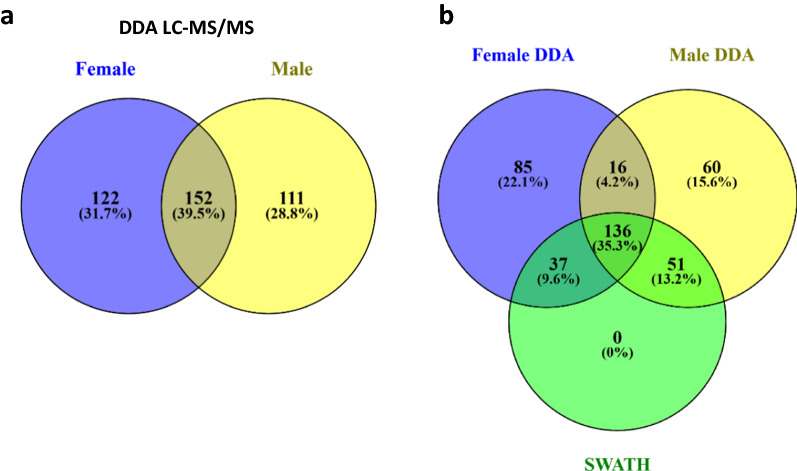
Venn diagrams depicting number and overlap of salivary proteins in female and male saliva detected by DDA LC-MS/MS (**a**) and DDA and SWATH-MS (**b**)

The spectrometric and identification data extracted from these libraries are summarised in Fig. 3 and Additional file 1: Table S1. Up to 470 and 469 protein hits were detected in female and male saliva, respectively. After eliminating the hits to non-annotated sequences in the *O. erraticus* sialotranscriptome database and the redundant identifications, we obtained two filtered lists of 274 and 263 non-redundant proteins from females and males, respectively. Of these, 152 proteins were found to be common to both sexes and 122 and 111 were found to be unique to males and females, respectively, which equates to 385 unique salivary proteins (Fig. 3a). This result indicates that male and female saliva has a different protein composition, since apparently only 39.5% of the identified proteins are expressed by both sexes. Regarding the reference spectral library, up to 639 protein hits were obtained. After removing hits to non-annotated proteins and redundant identifications, we obtained a list of 380 unique annotated proteins (Additional file 1: Table S1). This library was used as a reference for the analysis of SWATH-acquired data. Merging these three identification lists resulted in 387 unique proteins identified in the saliva of *O. erraticus* adult ticks.

The 387 characterised proteins, including their quantification by SWATH-MS and their functional annotation and classification, are shown in Additional file 5: Table S2. The number of proteins identified herein in *O. erraticus* saliva is comparable to those found in recent proteomic studies of the saliva of other argasid ticks and reflects a similar level of complexity and functional redundancy [25, 28, 29, 37]. These results represent a great improvement in the proteomic identifications of salivary proteins for this species, as only six salivary proteins of *O. erraticus* had been identified up to this point, in a earlier proteomics study using bi-dimensional gel electrophoresis and MALDI-TOF MS/MS [47].

### Proteins detected by SWATH-MS

While DDA MS methods are based on the random selection and fragmentation of a fixed number of peptide precursors, which are generally the most intense peptide ions, in SWATH-MS data acquisition, all ionised peptides of a given sample that fall within a specified mass range (*m*/*z*) are fragmented in a systematic and unbiased fashion using rather large precursor isolation windows [42]. Several studies have shown that SWATH-MS may outperform DDA LC–MS/MS by increasing the sensitivity and reproducibility of protein and peptide identification across multiple replicates [25, 48, 49]. Accordingly, we wanted to determine whether SWATH-MS might detect and quantify a higher number of proteins in *O. erraticus* male and female saliva than DDA LC–MS/MS, which would also allow protein expression levels to be compared between sexes.

Thus, individual female and male saliva samples were analysed by SWATH-MS, and its performance was compared to that of DDA LC–MS/MS. In female saliva, 274 and 224 proteins were identified by DDA and quantified by SWATH, respectively (Additional file 1: Table S1; Additional file 5: Table S2). Up to 173 of these proteins were detected by both methods, 101 exclusively by DDA and 51 exclusively by SWATH (Fig. 3b). In male saliva, 263 and 224 proteins were identified by DDA and quantified by SWATH, respectively (Additional file 1: Table S1; Additional file 5: Table S2). Up to 187 male proteins were detected by both methods, 76 exclusively by DDA and 37 exclusively by SWATH (Fig. 3b).

Although SWATH-MS did not outperform DDA-MS in terms of the total number of identified proteins (224 vs. 387), it is interesting to note that SWATH-MS detected more proteins common to/expressed by both sexes than did DDA-MS, namely 224 vs. 152 (Fig. 3a). This represents a 47.4% increase in the identification of common proteins and indicates that at least 57.9% (224/387) of all proteins identified in *O. erraticus* saliva are expressed by both sexes, which reduces the qualitative differences observed by DDA-MS and rather suggests quantitative differences instead.

Differences in the protein composition of saliva between females and males is well established in slow-feeding ixodid ticks [40, 50] and can be related to differences between sexes in terms of their feeding behaviour, anatomy and functions of the salivary glands [51–53]. In contrast to ixodids, adult argasid ticks are typically are fast-feeders, there are no anatomical or functional differences in the salivary glands between sexes and the feeding behaviours of females and males are similar, with both sexes ingesting equivalent amounts of blood relative to their body weight during a similar time interval, usually < 1 h. This means that female and male argasid ticks face the same host defensive responses; as such, it could be hypothesised that both sexes would use the same repertoire of anti-defensive salivary proteins to complete feeding [43].

Contrary to this hypothesis, the different band patterns shown in Fig. 2 and the different set of proteins identified by LC–MS/MS in female and male saliva suggest qualitative differences in the protein composition of *O. erraticus* saliva between both sexes. However, it must be noted that the *O. erraticus* sialotranscriptome used in the current study as a reference database for protein identification was obtained from female salivary glands only, leading us to suppose that: (i) the majority of proteins identified in the present study, including those detected in males only, would also most likely be expressed in female saliva; (ii) the proteins detected in the current study only in females would likely be proteins exclusively expressed by females; and (iii) the salivary proteins exclusively expressed by males, if any, would not have been detected in this study. Consequently, most of the differences observed between the sexes in the present study are most likely due to quantitative differences in expression, and to a lesser extent due to the genuine absence/presence of concrete proteins in one or other sex. The quantitative results of SWATH-MS also lend support to this hypothesis (as shown in later sections).

Differences in the protein composition of saliva between the sexes in other argasid species were reported by Díaz-Martín et al. [43] for *Ornithodoros moubata*, and recently confirmed at the quantitative level by Oleaga et al. [25]. The factors underlying these differences in argasids remain unknown, but they are possibly related to the post-feeding processing of the ingested blood and/or to attraction and mating [43, 54].

### Functional annotation and classification of the proteins identified in the saliva of *O. erraticus*

The 387 unique proteins identified in the saliva of *O. erraticus* adults were functionally annotated and characterised using the GO terms and cross-references in the InterPro, Pfam and Panther databases associated with UniProt IDs (Additional file 5: Table S2).

As many as 223 proteins were assigned GO terms; these included 121 cellular components, 114 molecular functions and 208 biological processes, all of which were visualised using the Web Gene Ontology Annotation Plot (WEGO) [55]. The classification of these proteins according to cellular component, molecular function and biological process, using level 2 GO terms, is shown in Additional file 6: Figure S1 . The cellular components were classified into 13 categories, of which the more abundantly represented were, jointly, cell and cell part (*n* = 136), organelle and organelle part (*n* = 59), membrane and membrane part (*n* = 58) and extracellular region and extracellular region part (*n* = 48). Classification by molecular function resulted in eight categories, with the most abundantly represented categories by far being those of catalytic (*n* = 112) and binding activity (*n* = 107); the remaining categories were noticeably less represented. The classification of biological processes resulted in 18 categories. The two most abundant categories were cellular processes (*n* = 82) and metabolic process (*n* = 78); again, the remaining categories were markedly less represented. This GO distribution is similar to those recently reported for the sialotranscriptomes of *O. erraticus* and *O. moubata* females [24, 26].

The functional classification of the 387 proteins according to the categories reported by Kim et al. [41] resulted in 24 functional groups and families (Table 1; Additional file 5: Table S2). Among these, the most numerous (i.e. with a higher number of proteins) were the proteins involved in metabolic processes (*n* = 71), proteases (*n* = 44), protease inhibitors (*n* = 32), lipocalins (*n* = 25), antioxidants (*n* = 22) and proteins with unknown function (*n* = 59), with the latter category representing 15.2% of the proteins identified. Comparison of the sexes showed that lipocalins, antioxidants and proteins involved in the metabolism of carbohydrates, energy and nucleic acids were more numerous in males than in females, while the remaining categories were either more numerous in females or equally numerous in both sexes (Table 2). Typically, these functional groups and families are also the most abundantly represented in the sialomes of the soft and hard tick species analysed to date [28, 29, 37].

**Table 1:** Number of proteins identified in saliva of *Ornithodoros erraticus* females and males by LC–MS/MS and SWATH-MS as classified in 24 different functional groups and families

Classification	LC–MS/MS			SWATH (female + male)
	Adult pool	Female	Male	
Antimicrobial	2	2	2	2
Antioxidant/detoxification^a^	22	12	19^a^	16
Cytoskeletal	17	14	11	10
Extracellular matrix	13	11	5	6
Glycine rich	3	3	3	3
Heme/iron binding	11	10	9	10
Immune related	12	10	8	7
Lipocalin^a^	25	18	20^a^	16
Metabolism, amino acids	1	1	1	1
Metabolism, carbohydrates^a^	22	8	20^a^	19
Metabolism, energy^a^	16	5	14^a^	7
Metabolism, lipids	14	10	10	10
Metabolism, nucleic acids^a^	18	11	15^a^	6
Nuclear regulation	16	13	7	4
Protease	44	31	29	24
Protease inhibitor	32	28	23	26
Proteasome machinery	8	4	4	1
Protein modification	9	5	5	5
Protein synthesis	5	4	1	1
Signal transduction	11	9	4	5
Transcription machinery	2	1	1	–
Transporter/receptors	22	16	14	11
Transposon element	3	2	2	1
Unknown function	59	46	36	33
Total	387	274	263	224

Additional details can be found in Additional file 1: Table S1

^a^Functional groups and families containing a higher number of proteins in male than in female saliva

**Table 2:** Proteins differentially expressed between female and male saliva

Functional category	Accession	Description	Mean signal peak area × 10^3^ (*n* = 3)—female	Mean signal peak area × 10^3^ (*n* = 3)— male	Fold change (female/male)	*P*-value^a^
Antimicrobial	ACB70385	Hebreain-like protein	95.60	313.28	0.31	1.31E−02
Antioxidant/detoxification	B7PHC3	Carbon–nitrogen hydrolase, putative	2086.45	320.63	6.51	3.10E−02
	PRD32710	Mitochondrial amidoxime reducing component 2	755.23	160.62	4.70	2.83E−03
	B7PQF4	Sulfotransferase, putative	30.34	73.69	0.41	1.86E−02
	B7QGH2	Glutathione *S*-transferase, putative	161.86	495.61	0.33	6.81E−03
	ABI52820	Superoxide-dismutase	99.49	325.97	0.31	1.88E−02
	AWM72026	Catalase 1	716.85	2,616.34	0.27	2.61E−02
	RZF47230	Hypothetical protein LSTR_LSTR004939	84.68	684.44	0.12	3.09E−06
Cytoskeletal	ABP01547	beta-Actin	631.00	966.00	0.65	4.54E−02
Extracellular matrix	AAS01023	Mucin/peritrophin-like protein precursor	81.49	0.05	1,644.20	4.72E−02
	XP_015907551	Laminin subunit beta-1 isoform X1	123.26	0.24	524.48	4.34E−02
	B7Q2R9	Cell adhesion molecule, putative	14.83	51.41	0.29	2.66E−03
Glycine rich	B7Q6J2	Glycine proline-rich secreted protein, putative	2406.02	748.22	3.22	3.30E−02
Heme/iron binding	AXP34687	Vitellogenin-1	6447.77	4.84	1333.20	3.47E−02
	AJR36491	Hemelipoglyco-carrier protein CP3	3552.23	8.50	418.01	2.25E−03
	ISCW021710-PA	CP3	866.12	25.82	33.54	8.62E−05
	AXP34690	Vitellogenin-B	1595.72	357.35	4.47	3.18E−02
Immune related	B7Q4R4	Double sized immunoglobulin G binding protein A	3993.51	779.57	5.12	1.32E−02
	DAA34752	Ixodegrin	44.01	135.43	0.32	3.96E−02
	AAM54048	Savignygrin	92.22	1021.83	0.09	4.38E−06
Lipocalin	ABI52661	Lipocalin	785.86	196.68	4.00	1.10E−04
	ACB70384	Salivary lipocalin	2161.01	667.58	3.24	3.86E−02
	ACB70386	Salivary lipocalin, partial	94.71	31.31	3.03	2.93E−02
	ADI60053	Savicalin	2749.89	2389.58	1.15	1.60E−04
	ABR23414	Moubatin-like 3	203.26	406.82	0.50	6.81E−03
	ABI52654	Monotonin	1149.91	2585.77	0.44	7.50E−04
	B7QAQ5	Putative uncharacterised protein (fragment)	28.73	99.58	0.29	2.29E−03
	ABR23394	Truncated salivary lipocalin, partial	15.89	84.78	0.19	2.24E−03
	ABR23399	Moubatin 1-like 2	466.29	6239.00	0.07	2.10E−03
	ABR23443	Salivary secreted lipocalin	35.76	1031.18	0.03	2.63E−03
	ABR23457	Moubatin-like 5	12.16	5932.07	0.00	1.12E−03
Metabolism, amino acids	TDG38496	Hypothetical protein AWZ03_015082	486.14	3401.02	0.14	1.70E−04
Metabolism, carbohydrates	B7QBY6	Glyoxalase, putative	41.51	96.62	0.43	1.22E−02
	XP_013777447	Glucose-6-phosphate isomerase	271.80	808.75	0.34	7.15E−03
	DAA34560	Malate dehydrogenase	81.64	330.49	0.25	7.34E−06
	B7PLL4	Fructose-1,6-bisphosphatase, putative	128.30	534.11	0.24	1.21E−03
	B7Q0R0	Phosphoglycerate mutase, putative	238.67	1208.54	0.20	2.08E−03
	AIW65719	Phosphoglucomutase, partial	15.03	89.67	0.17	3.46E−05
	ADD91327	Enolase	474.65	2936.96	0.16	2.40E−04
	ISCW015616-PA	3-Phosphoglycerate kinase, putative	437.28	2707.91	0.16	1.70E−04
	B7Q3K7	Tpi description	240.42	1591.72	0.15	3.25E−05
	ISCW020197-PA	Pyruvate kinase, putative	273.95	3468.12	0.08	1.74E−03
	ASV64058	Fructose-1,6-bisphosphate aldolase	917.27	15,028.55	0.06	1.20E−04
Metabolism, energy	KDR16306	Glycogen debranching enzyme, partial	109.61	280.36	0.39	7.04E−03
	ISCW018700-PA	Glyceraldehyde 3-phosphate dehydrogenase, putative	659.81	5964.82	0.11	1.30E−03
Metabolism, lipids	KFM77310	Apolipoprotein B-100, partial	314.75	3.03	103.96	1.56E−02
	ACB70350	Phospholipase A2, partial	264.73	67.48	3.92	1.39E−02
	AGJ90343	Phospholipase A2	3359.95	3070.84	1.09	2.13E−03
	ABI52805	Phospholipase A2, partial	328.61	812.17	0.40	3.45E−02
	ABR23453	Phospholipase A2	7.81	21.19	0.37	1.15E−02
	CAX51408	Hypothetical protein, partial	39.25	113.37	0.35	2.93E−02
	XP_015915960	Phosphatidylinositol transfer protein alpha isoform	2.92	45.86	0.06	4.20E−03
Metabolism, nucleic acids	B7PCV9	Lysosomal acid phosphatase, putative	2148.64	1148.01	1.87	5.67E−03
	ABS30897	5′-Nucleotidase/putative apyrase isoform 2 precursor	270.86	191.26	1.42	4.18E−03
	AGJ90350	Apyrase	232.08	234.82	0.99	3.72E−02
	B7PJJ3	Adenosine deaminase, putative	95.83	391.27	0.24	6.30E−04
Nuclear regulation	AHN53412	Histone H2B	7.83	22.10	0.35	3.12E−02
	XP_017008549	Histone H4-like, partial	2.16	33.42	0.06	4.00E−04
Protease	B7PF28	Longipain, putative	63.26	0.13	500.99	2.57E−02
	AMO02552	Carboxypeptidase Q	86.22	1.90	45.40	4.53E−02
	ABO26562	Cathepsin L-like cysteine protease	659.14	131.99	4.99	1.26E−02
	ISCW004835-PA	Coagulation factor precursor, putative	294.51	60.38	4.88	2.15E−02
	XP_013774340	Carboxypeptidase E-like	270.53	117.03	2.31	3.47E−02
	B7PJ51	Serine carboxypeptidase, putative (Fragment)	1492.78	648.27	2.30	1.90E−03
	XP_019696599	Neprilysin-1	97.36	43.48	2.24	2.21E−02
	ABI52714	Metalloprotease, partial	450.02	211.19	2.13	3.26E−02
	B7QLA0	Riddle, putative (fragment)	1308.48	3209.73	0.41	4.00E−03
Protease inhibitor	ABI94058	Serpin-8 precursor	49.66	2.44	20.35	2.89E−03
	XP_015907865	Hemocytin	7161.53	1838.73	3.89	4.29E−02
	B7P6X9	Hemolectin, putative (fragment)	1431.00	1045.88	1.37	3.44E−02
	KFM64508	Hemocytin, partial	631.61	667.75	0.95	4.25E−02
	AAS01022	Putative thyropin precursor	2472.52	4434.56	0.56	1.26E−02
	P83516	Chymotrypsin-elastase inhibitor ixodidin	382.07	759.34	0.50	2.26E−03
	XP_022121224	Hemocytin	70.57	173.91	0.41	4.28E−02
	XP_023217368	Leukocyte elastase inhibitor A-like isoform X2	19.51	52.26	0.37	4.18E−03
	XP_022255312	Hemocytin-like	22.22	62.06	0.36	7.08E−03
	ACB70299	Ixodidin	16.77	89.06	0.19	1.88E−03
	AAS01021	Cystatin precursor	167.09	1216.49	0.14	1.15E−03
	XP_020288964	Chymotrypsin inhibitor-like	195.85	1493.56	0.13	1.73E−02
	ACF57858	Chymotrypsin inhibitor precursor	233.56	3678.45	0.06	1.37E−03
Protein modification	B7PWF5	Heat shock protein 20.5, putative	113.92	5.08	22.43	4.59E−02
	B7PAR6	Heat shock protein, putative	222.48	1285.43	0.17	2.46E−03
Signal transduction	XP_021001474	Sushi, von Willebrand factor type A, EGF and pentraxin domain-containing protein 1	158.76	8.41	18.89	7.25E−03
Transporter/receptors	AGQ57038	Vitellogenin receptor	651.87	25.70	25.37	3.94E−06
	XP_015908566	Low-density lipoprotein receptor-related protein 2	107.78	53.93	2.00	1.88E−03
	XP_018014210	Very low-density lipoprotein receptor-like	783.98	924.47	0.85	1.22E−02
	B7PL51	ML domain-containing protein, putative	93.47	491.13	0.19	4.30E−04
Unknown function	XP_023240426	Synaptogenesis protein syg-2-like	137.70	1.41	97.65	3.88E−03
	ACB70374	Putative salivary secreted protein, partial	1704.00	91.42	18.64	2.08E−02
	ABI52697	7 cysteine domain	213.20	134.32	1.59	1.96E−03
	ABR23379	Salivary basic tailless protein	1124.06	1987.85	0.57	9.02E−05
	XP_023213207	Transmembrane protein 241-like	70.75	178.42	0.40	9.99E−03
	AAS94230	Unknown secreted protein PK-26 precursor	13,381.20	43,089.64	0.31	2.25E−02
	ABR23361	Acid tail salivary protein	14.64	53.69	0.27	3.47E−03
	B7PLB9	Secreted protein, putative	502.91	4091.22	0.12	6.10E−04
	ACB70369	Acid tail salivary protein	11.20	151.34	0.07	9.36E−05
	XP_022900755	S-phase kinase-associated protein 1	189.20	5274.83	0.04	2.50E−04

^a^*P* < 0.05

### Quantification by SWATH-MS of the proteins identified in female and male saliva

As already noted, SWATH-MS is a type of DIA method of analysis used to evaluate quantitatively complex samples with high reproducibility [42].

Using this technique, we detected and quantified 224 proteins in the saliva of both female and male ticks, which were later classified into 23 functional groups and families (Table 1; Additional file 7: Table S3), most of which coincide with the groups and families more abundantly represented in the sialotranscriptome of *O. erraticus* females [26].

**Fig. 4:**
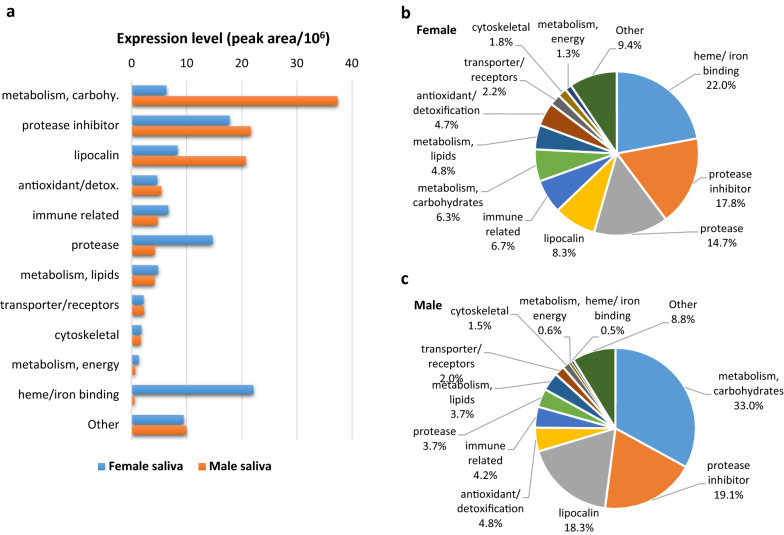
Expression levels of the identified proteins classified by functional groups and families. The expression level was calculated as the average spectral signal peak area in female (F1, F2, F3) and male (M1, M2, M3) saliva samples. **a** Comparative expression level between female and male saliva. **b**, **c** Pie charts showing the percentage of abundance of each functional group/family in the saliva of females (**b**) and males (**c**)

The expression levels of these protein groups and families in the saliva of both sexes, calculated as the average spectral signal peak area for samples F1, F2 and F3 or M1, M2 and M3, are shown in Additional file 7: Table S3 and summarised in Fig. 4. The 33 proteins with unknown function have been excluded from the charts, and the groups containing ≤ 6 proteins (metabolism of nucleic acids and amino acids, protein modification, glycine rich, antimicrobial, extracellular matrix, nuclear regulation, signal transduction, proteasome machinery, transposon element and protein synthesis) have been merged into one group named “Other”. Clear differences in protein composition at the quantitative level between female and male saliva are shown on Fig. 4.

In female saliva, the most abundantly expressed functional category was heme/iron binding, with these proteins representing 22% of the protein mass in this fluid (Fig. 4a, b). This functional category was followed by—in terms of abundance—the functional categories of protease inhibitors (17.8%), proteases (14.7%), lipocalins (8.3%) and immune-related (6.7%). In contrast, the proteins involved in carbohydrate metabolism represented the most abundant functional category in male saliva (33.0%), followed by the categories of protease inhibitors (19.1%) and lipocalins (18.3%) (Fig. 4c).

#### Heme/iron binding

The heme/iron binding group included 10 proteins: five vitellogenins (Vgs) and five hemelipoglyco-carrier proteins (CPs), with Vgs being the most abundant as they accounted for 73.6% of the protein mass of this group (Table 1; Fig. 4; Additional file 7: Table S3). The iron-containing heme group is required for normal tick physiology, including egg embryogenesis and reproduction. Since ticks do not have a heme biosynthetic pathway, they must obtain heme from host blood. However, as free heme is toxic to ticks, heme metabolism and management must be tightly regulated [56, 57]. Heme-binding proteins, such as Vgs and CPs, take part in the removal and detoxification of free heme excess, as well as in lipid transport and storage [56–58]. Additionally, Vgs are precursors of vitellin, a protein that is essential for egg development and oviposition [59], which could explain the high abundance of vitellogenins in female saliva compared to male saliva, where these proteins represented only 0.45% of the total protein mass (Additional file 7: Table S3). Vgs were originally thought to be synthesised in the midgut and fat body only. However, Vg mRNA has been recently found in the salivary glands of *Rhipicephalus bursa*, *O. moubata* and *O. erraticus*, where it is up-regulated in response to blood-feeding [24, 26, 31]. Moreover, the Vg protein has been abundantly detected in the saliva of *A. americanum* [41] and *O. moubata* [25], consistent with our current results. These findings indicate that heme-binding Vg-like proteins are indeed synthesised in the salivary glands and abundantly secreted into the host with tick saliva, where they likely play relevant functions in tick feeding; for example as anti-inflammatory agents, by reducing the concentration of free heme at the feeding lesion, which in turn diminishes the heme potential to promote inflammation as well as heme cytotoxicity [31, 41], or even as antioxidants and transporters of cholesterol, phospholipids and fatty acids [60]. The up-regulation of Vg-like proteins upon feeding, their abundance and functions in tick saliva, and the recognised high immunogenicity of tick Vgs [31] make this group of proteins interesting targets for tick vaccines.

#### Protease inhibitors

Most of the defensive responses that hosts deploy upon tick bites, including haemostasis, inflammation and immunity, are mediated by proteases, particularly serine and cysteine proteases [61, 62]. Accordingly, tick saliva contains an abundance of protease inhibitors, mainly serine and cysteine protease inhibitors, which counteract host defences and facilitate blood ingestion [63, 64].

Up to 26 protease inhibitors were detected and quantified by SWATH-MS in female and male saliva samples (Table 1; Additional file 7: Table S3). These represented 17.8% and 19.1% of the protein mass in female and male saliva, respectively (Fig. 4b, c) and were the second most abundant functional category in both sexes. Twenty-three of these protease inhibitors were serine protease inhibitors and the remaining three were cysteine protease inhibitors or cystatins.

Among the serine protease inhibitors, we found Kunitz domain inhibitors, trypsin inhibitor-like cysteine rich domain (TIL) inhibitors and serpins. Kunitz-type inhibitors are abundant in tick sialomes/saliva and include numerous anticoagulants that inhibit different proteases in the coagulation cascade, mainly thrombin and factor Xa [37, 40, 41, 65]. TIL inhibitors form a family commonly found in blood-feeding insects and tick sialomes, which includes chymotrypsin, elastase and trypsin inhibitors. Some TIL inhibitors are known to act as anti-inflammatory agents and others behave as antimicrobial peptides [66, 67]. Serpins are also abundantly detected in tick sialomes/saliva, where they play a role as immunomodulators, anti-inflammatory agents and inhibitors of platelet aggregation and blood coagulation [41, 63, 68]. Two types of cystatins have been reported in ticks: type 1 cystatins, which are intracellular and involved in the intracellular digestion of haemoglobin and developmental processes, and type 2 cystatins, which are typically secreted to saliva, where they act as immunomodulators in the tick–host relationship [63].

Hemocytin was the most abundant protease inhibitor in female saliva, representing 44.3% of the protein mass in this category (Additional file 7: Table S3). Hemocytin is a large modular glycoprotein, orthologue of the human von Willebrand factor, and contains several serine inhibitor TIL domains and several domains homologous to coagulation factor VIII. Hemocytin was first described in the silkworm *Bombyx mori* and is functionally related to the insect immune response to invading pathogens through mechanisms such as haemolymph coagulation, haemocyte aggregation and nodule formation [69, 70]. Hemocytin has been also detected in tick genomes [71], and although its functions in ticks remain to be elucidated, it could participate in the defence against pathogens ingested with blood.

On the other hand, a putative thyropin was the most abundantly detected protease inhibitor in male saliva (up to 20.5% of the protein mass in this category) and the second most abundant in female saliva (13.9%) (Additional file 7: Table S3). Thyropins are cysteine protease inhibitors that contain thyroglobulin type-1 (Thyr-1) domains and inhibit either cysteine or cation-dependent proteases [72]. Thyropins have been found in the sialomes and mialomes of several hard and soft ticks [37, 73, 74]. Their function in ticks remains unknown, but it has been suggested that they may act as immune modulators by regulating endosomal cathepsins, cysteine proteases involved in antigen processing by antigen-presenting cells [73].

#### Proteases

Up to 24 proteases, including serine, cysteine, metallo- and aspartic proteases, were quantified by SWATH-MS in *O. erraticus* saliva samples, which represented 15% and 4% of the protein mass in female and male saliva, respectively (Table 1; Fig. 4). Serine proteases were the most abundant proteases in female saliva, accounting for 51.1% of the protein mass of this group, followed by cysteine (37%), metalloproteases (10%) and aspartic proteases (1.8%). In contrast, in male saliva, the most abundant proteases were cysteine proteases (46.9%), followed by serine (40.1%) metalloproteases (12.9%) and aspartic proteases (0.2%) (Additional file 7: Table S3).

Serine proteases are commonly found in salivary glands and saliva from argasid and ixodid ticks [24, 26, 37, 41]. Salivary serine proteases of ticks are thought to regulate host defensive mechanisms at the tick bite site, such as blood clotting and fibrinolysis, matrix remodelling, inflammation and innate immunity, all of which may facilitate tick blood-feeding [39, 73, 75].

Tick cysteine proteases, including L and B cathepsins and longipain, are mostly expressed in the midgut and involved in blood meal digestion, embryogenesis and pathogen transmission [76–78]. However, the abundance of cysteine proteases found in *O. erraticus* saliva suggests that they might be also playing a role in tick feeding. Recently, a cathepsin L from *Rhipicephalus microplus* was shown to impair thrombin-induced fibrinogen clotting via a fibrinogenolytic activity, contributing to maintain blood fluidity of the ingested blood [62]. If the cysteine proteases found in *O. erraticus* saliva play a similar anti-clotting role, they might also contribute to maintaining blood fluidity, thereby helping ticks to ingest the blood meal. Notably, the most abundant cysteine protease in *O. erraticus* saliva was a gamma-glutamyl hydrolase-like isoform X1 (XP_013781036). Gamma-glutamyl hydrolase (GGH) is a ubiquitously expressed lysosomal enzyme that regulates intracellular folate metabolism for cell proliferation, DNA synthesis and repair [79]. GGH progressively removes gamma-glutamyl residues from poly-gamma-glutamyl forms of folic acid to yield folic acid and free glutamate. The abundance of this housekeeping enzyme in *O. erraticus* saliva suggests that it might play an additional extracellular function at the host–parasite interface, as has already been observed for other intracellular housekeeping enzymes, such as the salivary phospholipase A2 and an enolase of *O. moubata*, which act as anti-inflammatory and anti-haemostatic agents [80–82]. Intracellular housekeeping proteins are usually found as components of soft and hard tick saliva, where they can be secreted by unconventional mechanisms as predicted by the SecretomeP tool, such as apocrine secretion and/or secretion in microvesicles (exosomes) [25, 40, 43].

Tick metalloproteases are expressed in the midgut, ovary, salivary glands and saliva [37, 83, 84]. Salivary metalloproteases are quite abundant and diverse, playing varied functions related to modulation of the host defensive responses that can facilitate blood-feeding. For example, salivary metalloproteases may contribute to the formation of the feeding pool by degrading host extracellular matrix proteins at the tick bite site; metalloproteases also display anti-clotting and anti-inflammatory activity by degrading fibrinogen, fibrin and inflammatory mediators, and could even prevent host tissue repair because of their anti-angiogenic activity [40, 61, 74]. As a result of these activities, the immunoprotective potential of a *R. microplus* metalloprotease was analysed and shown to confer 60% protection against tick infestation, highlighting this metalloprotease as a potential candidate for an anti-tick vaccine [83]. Metalloproteases were found to be the most abundantly represented proteases in the *O. erraticus* sialotranscriptome [26] and in other tick sialomes [24, 37, 39, 40, 74]. However, in the *O. erraticus* saliva proteome, metalloproteases are only the third most abundant protease family, suggesting some additional post-transcriptional regulation, with neprilysins being the most numerous and abundantly represented metalloprotease group (Additional file 5: Table S2).

#### Lipocalins

Lipocalins constitute a large and diverse family of secreted proteins that bind to and transport small hydrophobic molecules. Lipocalins are abundantly represented in tick sialomes, where they contribute to evading the haemostatic, inflammatory and innate immune responses of the host, mainly as scavengers of biogenic amines and eicosanoids [24, 26, 37, 41, 43, 74].

In this study, we found that lipocalins were the fourth most abundantly represented protein family in female saliva (8.3% of the protein mass) and the third most abundant in male saliva (18.3% of the protein mass) (Fig. 4). Up to 16 lipocalins were quantified by SWATH-MS in both sexes (Additional file 7: Table S3), of which six belonged to the biogenic amine (histamine and serotonin) binding clade of lipocalins [85] and another four belonged to the moubatin-like clade [86]. Among the remaining lipocalins, the one annotated as savicalin (ADI60053) was remarkable by its high abundance.

Amine-binding lipocalins were the most abundant lipocalins in female saliva, accounting for up to 50.4% of the lipocalin mass versus the 8% represented by moubatin-like lipocalins (Additional file 7: Table S3). This abundance strongly suggests that it is very important to prevent the inflammatory reaction induced by the release of histamine at the tick-feeding lesion for *O. erraticus* females to be able to feed. On the contrary, moubatin-like lipocalins were the most abundant lipocalins in male saliva (60.8% of lipocalin mass), far outdistancing the amine-binding lipocalins that accounted for 18.0% of male lipocalin mass (Additional file 7: Table S3). The moubatin clade includes inhibitors of platelet and neutrophil aggregation, which act by scavenging thromboxane A2 (TXA2) and leukotriene B4 (LTB4), and inhibitors of complement activation, which sequester the C5 component [86–88]. The high abundance of moubatin-like lipocalins in male saliva suggests that blocking host haemostasis and innate immunity would be more important for *O. erraticus* males to be able to feed than preventing the histamine-mediated inflammatory reaction at the feeding lesion. Savicalin was also quite abundant in the *O. erraticus* saliva, accounting for 32.9% and 11.5% of the female and male lipocalin mass, respectively. Savicalin was first described in the haemocytes, midgut and ovaries of *Ornithodoros kalaharensis* [89] and more recently in the sialotranscriptome of *O. erraticus* [26]. Savicalin is upregulated in the midgut and ovaries after feeding and in haemocytes after bacterial challenge, suggesting its involvement in tick development and antimicrobial defence [89]. The potential function of savicalin in tick salivary glands and saliva is currently unknown, but it might be related to protection against pathogens acquired during feeding.

In contrast to our results, lipocalins were found to be by far the most abundant proteins in the saliva proteome of *O. moubata* [25, 43] and *R. microplus* [90], but marginal in the proteome of salivary glands of *Hyalomma dromedarii*, where they only accounted for 0.8% of the secreted proteins in both sexes [40].

#### Immune-related proteins

Seven immune-related proteins were quantified by SWATH-MS, accounting for 6.7% and 4.2% of the protein mass in the female and male saliva, respectively (Table 1; Fig. 4). The most abundant among these were an immunoglobulin G binding protein A, a cysteine-rich venom protein and two orthologues of savignygrin and ixodegrin (Additional file 7: Table S3).

Immunoglobulin-binding proteins (IGBPs) are used by ticks to evade the host immune system and the damage caused by host antibodies that are ingested with blood. Intact host antibodies taken up into the gut by a feeding tick pass through into the haemolymph, and in this way they can reach their antigen targets in internal organs. To prevent this immune mechanism, host immunoglobulins are bound, transported and finally excreted back to the tick-feeding site by IGBPs in haemolymph and salivary glands [91]. In ixodids, IGBPs are mainly expressed by males and secreted into the feeding site to help co-feeding females feed on blood and to remove antibodies from the tick itself, thereby preventing antibody-mediated damage. Our results show that IGPBs were approximately fivefold more abundant in female than in male saliva (Additional file 7: Table S3). This is an interesting finding because in contrast to ixodids, *O. erraticus* adults do not co-feed [6; personal observation], and there is no evidence that males assist females to feed or that females may have acquired proteins from male saliva. Accordingly, female and male *O. erraticus* will each have expressed and secreted their own salivary IGBPs. In this context, it seems logical that IGBPs would be more abundant in female than in male saliva because females ingest higher amounts of blood than males (average: 12 mg/female vs 3.5 mg/male), and thus higher amounts of host antibodies, which have to be neutralised. In argasids, IGBPs have been also detected in *O. moubata* male and female saliva [25], suggesting the conservation of these anti-defensive mechanisms among hard and soft ticks. Because of their function, IGBPs have been studied as potential vaccine candidates against ixodids, with partial success [92].

Cysteine-rich venom protein 1 belongs to the CAP [i.e. cysteine-rich secretory proteins (CRISPs), antigen 5 (Ag5) and pathogenesis-related 1 (PR-1) proteins] surperfamily of venomous proteins found in metazoans, including ticks [93, 94]. Snake venom CRISPs are the best-known CAPs, and some of them have been functionally characterised; they inhibit a number of ion channels and the growth of new blood vessels, acting as anti-angiogenic and vasodilator agents [95]. CRISPs in lamprey buccal gland secretions also act as inhibitors of ion channels and vasodilators, helping this haematophagous fish to feed [96], suggesting an evolutionarily conserved function for CRISPs that would facilitate haematophagy in ticks.

Savignygrin and ixodegrins are disintegrin-like inhibitors of platelet aggregation discovered in *O. kalaharensis* [97] and *Ixodes pacificus* [98], respectively. Disintegrins are peptides that have a RGD or KGD domain, which can bind to integrins and impede the interaction of integrins with their ligands, thereby blocking downstream events. In this way, the binding of disintegrins to integrin αIIbβ3 on activated platelets prevents fibrinogen–platelet interaction and inhibits platelet aggregation [99]. Platelet aggregation is the first step in the host haemostatic response and must be abrogated by ticks to feed; thus, anti-platelet aggregation agents, including disintegrins, are frequently found in tick sialomes [37]. Disintegrins can be interesting targets for tick vaccines, and some studies have explored the value of the recently discovered *O. moubata* mougrin [100] and of an *Ixodes ricinus* ixodegrin [101] as antigen candidates for anti-tick and pathogen transmission-blocking vaccines.

#### Carbohydrate metabolism

Nineteen proteins involved in the metabolism of carbohydrates were quantified by SWATH-MS (Table 1; Additional file 7: Table S3). These comprised the most abundant functional category in male saliva, accounting for 33.0% of the protein mass in this sex, yet they only represented 6.3% of the female saliva protein mass (Fig. 4b, c). Most of these 19 proteins (*n* = 14) are enzymes of the glycolysis and tricarboxylic acid (TCA) cycle pathways. Cytoplasmic glycolysis transforms glucose to pyruvate, which is transported to the mitochondria, converted in acetyl-CoA and later metabolised in the TCA cycle to yield reducing equivalents that enter the oxidative phosphorylation chain to produce ATP [102].

These enzymes are considered to be housekeeping proteins, and the potential extracellular functions they might play in the tick saliva and the host–parasite interface remain unknown, with some exceptions, such as enolase. Salivary enolase of *O. moubata* can bind host plasminogen and stimulate its activation to plasmin at the feeding lesion, promoting fibrinolysis and contributing to the prevention of blood clot formation [82]. A similar function could be presumed for the salivary enolase of *O. erraticus*, although this needs to be experimentally demonstrated.

The reason why these glycolytic enzymes are much more abundant in male saliva than in female saliva (Fig. 4) also remains unknown. It could be speculated that this difference would be a consequence of the additional functions that these enzymes might play in male saliva; these functions are currently unknown but may possibly be related to attraction, mating and spermatophore transfer [54, 103]. Interestingly, in another argasid species, *O. moubata*, the enzymes of the glycolysis pathway were also found to be more abundantly expressed in male than female saliva [25, 43], suggesting that this could be a conserved tendency in the *Ornithodoros* genus or even in the Argasidae family.

### Proteins differentially expressed between the sexes

Among the 224 common proteins quantified by SWATH-MS in female and male saliva, 97 were differentially expressed (*P* < 0.05) between the sexes (Table 2), of which 37 were overexpressed in females and 60 overexpressed in males. The signal peak areas of the differentially expressed proteins in each of the samples analysed were shown using a heat map after *Z*-score normalisation, using Euclidean distances. This heat map shows two main clusters comprising the F1–F3 samples and M1–M3 samples, which correspond to the saliva of females and males, respectively (Additional file 8: Fig. S2).

**Fig. 5:**
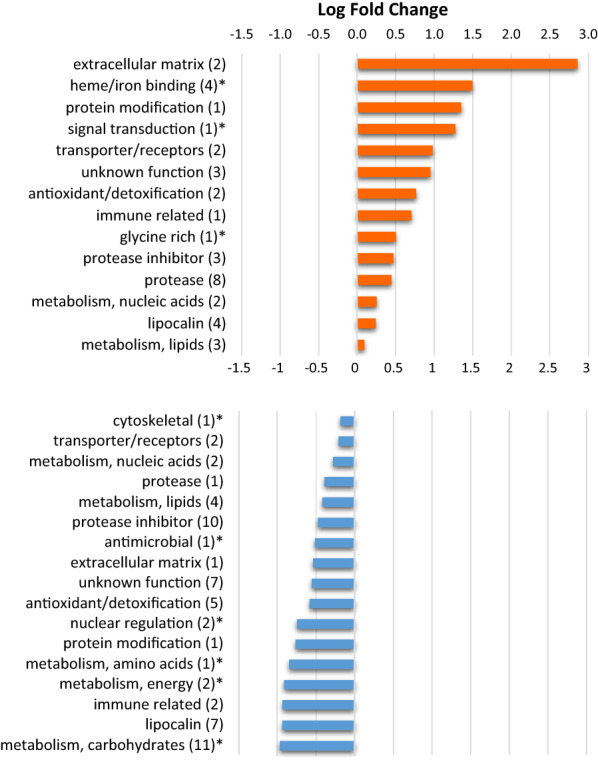
Functional categories containing differentially (*P* < 0.05) expressed proteins between female and male saliva. Fourteen and 17 categories contained overexpressed proteins in females (orange) and males (blue), respectively. Numbers in parentheses indicate the number of overexpressed proteins in each category. Asterisks indicate functional categories overexpressed only in females (*n* = 3) or in males (*n* = 6)

The 97 differentially expressed proteins were classified into functional categories according to Kim et al. [41], and the average log-fold change was calculated for each category and sex. Figure 5 shows that 14 and 17 categories contained overexpressed proteins in females and males, respectively. Eleven of these categories contained overexpressed proteins in both sexes; three categories (heme/iron-binding, signal transduction and glycine-rich proteins) contained proteins overexpressed only in females; and six categories (cytoskeletal, antimicrobial, nuclear regulation and metabolism of amino acids, energy and carbohydrates) contained proteins overexpressed only in males. In females, the overexpressed categories showing the highest fold change were extracellular matrix and heme/iron-binding, whereas the overexpressed categories showing the highest fold change in males were the metabolism of carbohydrates and lipocalins, in good agreement with the more abundant functional categories shown in Fig. 4.

Additional file 9: Fig. S3 represents the top 10 proteins that are differentially (*P* < 0.05) overexpressed in the saliva of female or male ticks. In agreement with the above-reported results, the top 10 differentially overexpressed proteins in females were three heme/iron-binding proteins (2 carrier proteins and 1 vitellogenin), two proteases (longipain and carboxipeptidase Q), two proteins of the extracellular matrix (mucin/peritrophin-like protein and laminin subunit beta-1), one apolipoprotein B-100 involved in lipid metabolism, one vitellogenin receptor and one protein with unknown function. In males, the top 10 differentially overexpressed proteins included three lipocalins (both of them moubatins), two glycolytic enzymes (pyruvate kinase and fructose-1,6-bisphosphate aldolase), one chymotrypsin inhibitor, one histone involved in nuclear regulation, a phosphatidylinositol transfer protein involved in lipid metabolism and two proteins of unknown function, one of them being an acid tail salivary protein (Table 2).

Mucins are glycoproteins usually found in ixodid and argasid tick sialomes, but their function in ticks has not been studied [24–26, 37, 41, 74]. Mucins are secreted into tick saliva and may function in tick feeding by coating and protecting the chitinous tick mouthparts and by interacting with proteins of the host extracellular matrix [75]. Additionally, indirect evidence in humans suggests that mucins might participate in the antimicrobial defence, as human mucins have been shown to encapsulate microbes [104].

Acid tail proteins, together with basic tail and tailless proteins, constitute a superfamily of tick-specific proteins abundantly found in the sialomes of both ixodid and argasid species [24–26, 37, 41, 74]. They are presumed to play important and specific roles at the tick–host feeding interface, but only a few members of this family have been functionally characterised as anti-coagulants [105–107] and specific complement inhibitors [108], while most of them have as yet unknown functions.

Taken together, these results of SWATH-MS show that at least 224 out of 387 of the salivary proteins identified in the current study are shared by both sexes, which significantly enhances the number of shared proteins identified by DDA LC–MS/MS (152 out of 387). However, these results show remarkable differences in the ratios of salivary proteins that males and females secrete in their saliva, which raises the question of the biological significance of these differences. It has been proposed that differences could be related to the post-feeding blood processing or attraction and mating [25, 43, 54], but this question remains unsolved.

## Conclusions

*Ornithodoros erraticus* has great medical and veterinary importance because it colonises anthropic environments and transmits the microbes that cause severe diseases affecting humans and domestic swine, such as TBRF and ASF. The prevention and control of these diseases would greatly benefit from the elimination of *O. erraticus* populations (and, possibly, from the elimination of other species of the *O. erraticus* complex) from at least the anthropic environments. Anti-tick vaccine development is a promising and sustainable alternative control strategy to the application of chemical acaricides. As tick saliva contains all of the bioactive molecules that the ticks use to abrogate host defences and successfully feed, decoding the protein composition of saliva will provide new antigen targets for anti-tick vaccines.

With this aim, we implemented a PIT approach to analyse the saliva of female and male *O. erraticus* using the recently obtained sialotranscriptome of *O. erraticus* females as a reference database for protein identification [26] and two MS techniques: conventional LC–MS/MS and quantitative label-free SWATH-MS.

This PIT approach demonstrated its usefulness for proteomics studies of *O. erraticus*, a non-model organism for which there are no genomic sequences available, as it allowed us to identify 387 non-redundant proteins in *O. erraticus* saliva. This is the first comprehensive proteome of the saliva of *O. erraticus* reported to date. The level of complexity and functional redundancy of the saliva proteome of *O. erraticus* were found to be similar to those reported for the saliva proteome of other argasids [25], although the similarity was not as high as that observed for the salivary proteome of ixodids [41].

SWATH-MS allowed the quantification of 224 shared proteins in both female and male saliva, among which 37 were significantly overexpressed in females and 60 in males. These results showed important quantitative differences between sexes in the expression of several functional categories of salivary proteins. Heme/iron-binding proteins, protease inhibitors, proteases, lipocalins and immune-related proteins were the most abundantly expressed categories in females, while glycolytic enzymes, protease inhibitors and lipocalins were the most abundantly expressed categories in males. The involvement of these proteins in tick feeding and host defence modulation was analysed in terms of their usefulness as vaccine target candidates. Additionally, we briefly discussed the factors underlying the differential expression of these proteins between the sexes.

These findings unveil novel salivary proteins and functions at the tick–host feeding interface and facilitate a better understanding of the physiology of feeding in *O. erraticus*, including possible sex-specific differences in the post-feeding processing of the ingested blood. The integration of this new proteomic knowledge and the former sialotranscriptomic data will drive a more coherent selection of salivary proteins as candidate antigens for the development of vaccines aimed at the control of *O. erraticus* populations, which may, in turn, contribute to the prevention of important medical and veterinary tick-borne diseases, such as TBRF and ASF.

## Supplementary Information

**Additional file 1: Table S1.** Number of proteins identified in *O. erraticus* female and male saliva by LC–MS/MS and SWATH-MS as classified in 24 different functional groups and families.

**Additional file 2: Dataset S1.** ProteinPilot report of female saliva digested in gel.

**Additional file 3: Dataset S2.** ProteinPilot report of male saliva digested in solution.

**Additional file 4: Dataset S3.** ProteinPilot report of the spectral library.

**Additional file 5: Table S2.** Salivary proteome. List of proteins identified and characterised in female and male saliva and in the spectral library by LC–MS/MS and SWATH-MS methods.

**Additional file 6: Figure S1.** Gene ontology (GO) distribution of *O. erraticus* saliva proteome. Level 2 GO terms of cellular components, molecular functions and biological processes were visualised using WEGO (Web Gene Ontology Annotation Plot).

**Additional file 7: Table S3.** List of salivary proteins quantified by SWATH MS including signal peak area, fold change (FC) for female versus male saliva, logFC and *P*-values.

**Additional file 8: Figure S2.** Heat map showing levels of differentially expressed proteins (*P* < 0.05) among female and male biological replicated samples, and hierarchical clustering showing two main clusters comprising F1–F3 samples and M1–M3 samples, corresponding to female and male saliva, respectively.

**Additional file 9: Figure S3.** Top 10 proteins that are differentially (*P* < 0.05) overexpressed in female (orange) or male (blue) saliva.

## Data Availability

The mass spectrometry proteomics data have been deposited to the ProteomeXchange Consortium via the PRIDE partner [46] repository with the dataset identifier PXD027367.

## Abbreviations

ABC: Ammonium bicarbonate
ACN: Acetonitrile
ASF: African swine fever
CP: Hemelipoglyco-carrier protein
DDA: Data-dependent acquisition
DIA: Data-independent acquisition
DTT: Dithiothreitol
FA: Formic acid
FDR: False discovery rate
GO: Gene ontology
IAM: Iodoacetamide
LC–MS/MS: Liquid chromatography–tandem mass spectrometry
PBS: Phosphate-buffered saline
PIT: Proteomics informed by transcriptomics
SDS-PAGE: Sodium dodecyl sulphate-polyacrylamide gel electrophoresis
SWATH-MS: Sequential window acquisition of all theoretical fragment ion spectra mass spectrometry
TBRF: Tick-borne human relapsing fever
TFA: Trifluoroacetic acid
Vg: Vitellogenins
