# Supplementary material for: A proteomics informed by transcriptomics insight into the proteome of Ornithodoros erraticus adult tick saliva
Source: Parasit Vectors. 2022 Jan 3;15:1. doi: 10.1186/s13071-021-05118-1 (PMC8722417; doi:10.1186/s13071-021-05118-1)
Supplement: Supplementary file 1 — Additional file 1: Table S1. Number of proteins identified in O. erraticus female and male saliva by LC–MS/MS and SWATH-MS as classified in 24 different functional groups and families. [file 13071_2021_5118_MOESM1_ESM.docx]

**Additional file 1. Table S1.** Identification performance of DDA LC-MS/MS analysis of female saliva digested in-gel, male saliva digested in-solution and spectral library, as well as of SWATH-MS analysis of individual samples of female and male saliva.

*Number of spectra, peptides and protein hits identified with FDR <1% to the *O. erraticus* sialotranscriptome database

|  | **Female pool (n=3) digested in gel** | **Male pool**  **(n=3)**  **digested in solution** | **Spectral library (female and male)** | **SWATH**  **(female and male)** |
| --- | --- | --- | --- | --- |
| No. of spectra | 7,763* | 4,717* | 12,503* | - |
| No. of peptides | 5,243* | 3,394* | 7,327* | - |
| Protein hits | 470* | 469* | 639* | 389 |
| Hits to non-annotated proteins (% of total protein hits) | 102 (21.7%) | 102 (21.7%) | 127 (19.9%) | 82 (21.1%) |
| Redundant identifications | 94 | 104 | 132 | 83 |
| Non-redundant annotated proteins | 274 | 263 | 380 | 224 |
