# Supplementary material for: A proteomics informed by transcriptomics insight into the proteome of Ornithodoros erraticus adult tick saliva
Source: Parasit Vectors. 2022 Jan 3;15:1. doi: 10.1186/s13071-021-05118-1 (PMC8722417; doi:10.1186/s13071-021-05118-1)
Supplement: Supplementary file 5 — Additional file 5: Table S2. Salivary proteome. List of proteins identified and characterised in female and male saliva and in the spectral library by LC–MS/MS and SWATH-MS methods. [file 13071_2021_5118_MOESM5_ESM.pptx]

## Slide 1
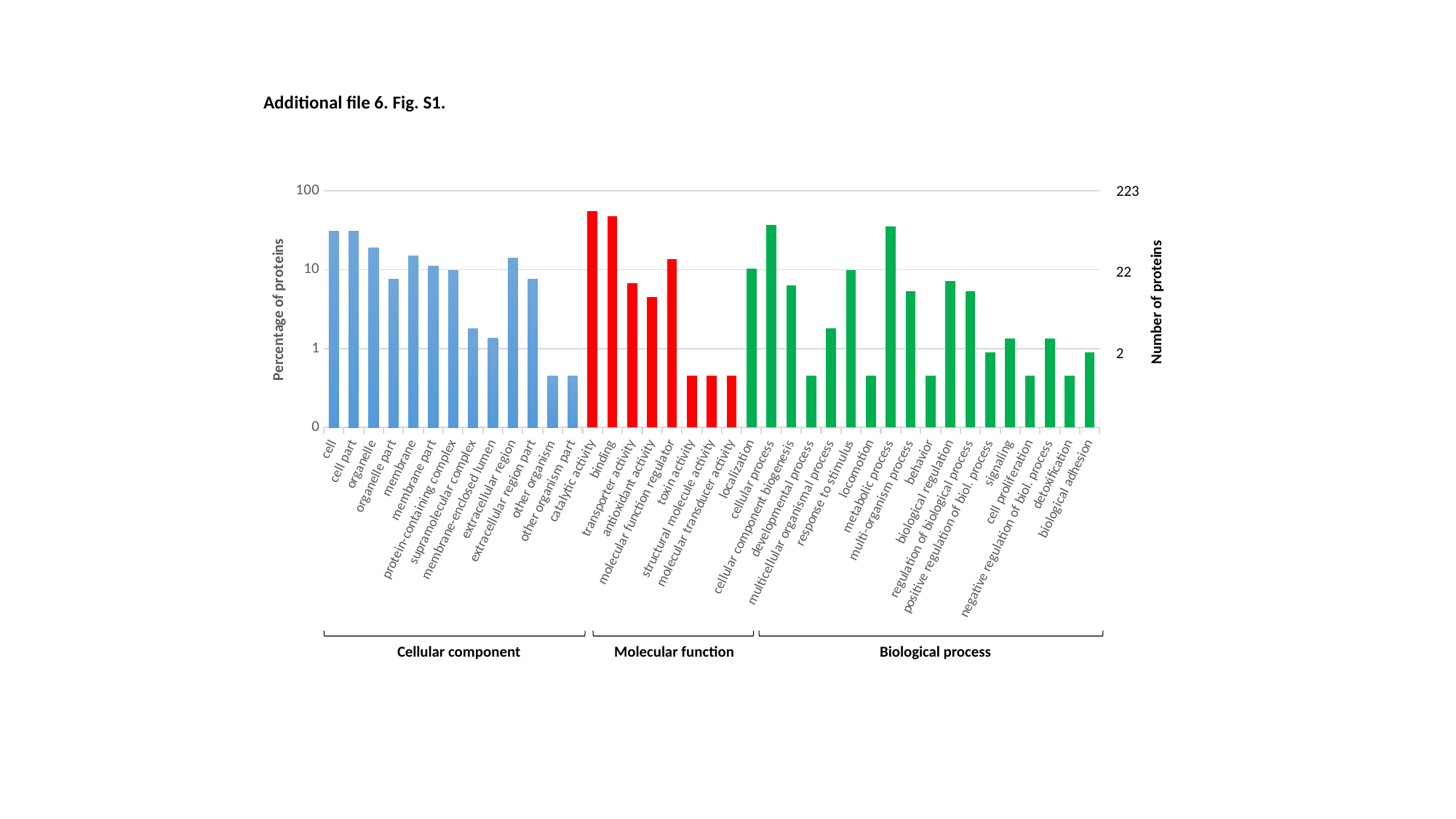

Additional file 6. Fig. S1.
### Chart
| Category | percentage of proteins |
|---|---|
| cell | 30.493273542600896 |
| cell part | 30.493273542600896 |
| organelle | 18.83408071748879 |
| organelle part | 7.623318385650224 |
| membrane | 14.798206278026905 |
| membrane part | 11.210762331838565 |
| protein-containing complex | 9.865470852017937 |
| supramolecular complex | 1.7937219730941705 |
| membrane-enclosed lumen | 1.345291479820628 |
| extracellular region | 13.90134529147982 |
| extracellular region part | 7.623318385650224 |
| other organism | 0.4484304932735426 |
| other organism part | 0.4484304932735426 |
| catalytic activity | 54.7085201793722 |
| binding | 47.98206278026906 |
| transporter activity | 6.726457399103139 |
| antioxidant activity | 4.484304932735426 |
| molecular function regulator | 13.452914798206278 |
| toxin activity | 0.4484304932735426 |
| structural molecule activity | 0.4484304932735426 |
| molecular transducer activity | 0.4484304932735426 |
| localization | 10.31390134529148 |
| cellular process | 36.771300448430495 |
| cellular component biogenesis | 6.278026905829597 |
| developmental process | 0.4484304932735426 |
| multicellular organismal process | 1.7937219730941705 |
| response to stimulus | 9.865470852017937 |
| locomotion | 0.4484304932735426 |
| metabolic process | 34.97757847533632 |
| multi-organism process | 5.381165919282512 |
| behavior | 0.4484304932735426 |
| biological regulation | 7.174887892376682 |
| regulation of biological process | 5.381165919282512 |
| positive regulation of biol. process | 0.8968609865470852 |
| signaling | 1.345291479820628 |
| cell proliferation | 0.4484304932735426 |
| negative regulation of biol. process | 1.345291479820628 |
| detoxification | 0.4484304932735426 |
| biological adhesion | 0.8968609865470852 |223
22
Number of proteins
2
Cellular component
Molecular function
Biological process
