# Supplementary figures and images for: A proteomics informed by transcriptomics insight into the proteome of Ornithodoros erraticus adult tick saliva
Source: Parasit Vectors. 2022 Jan 3;15:1. doi: 10.1186/s13071-021-05118-1 (PMC8722417; doi:10.1186/s13071-021-05118-1)

## Slide 1
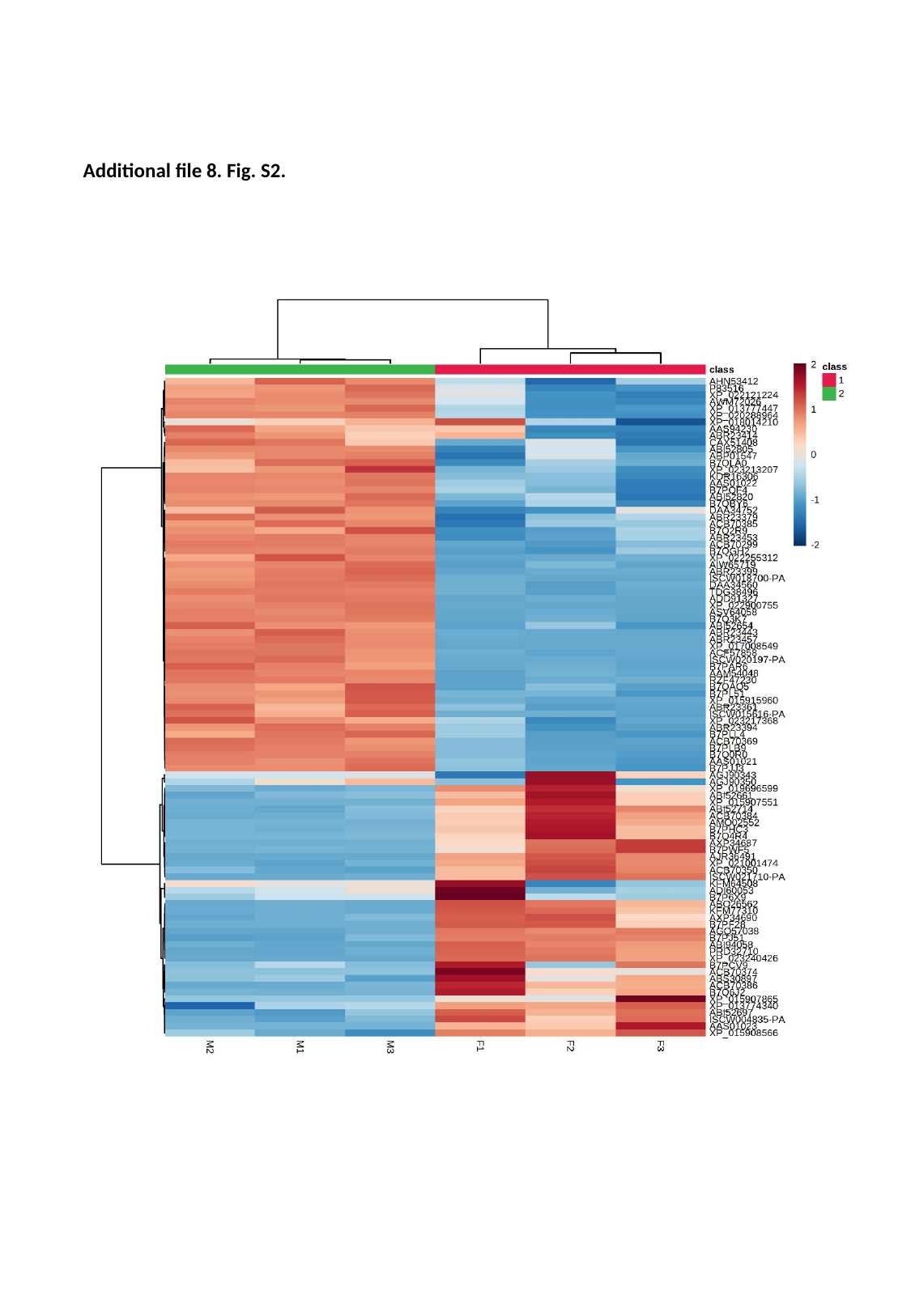

Additional file 8. Fig. S2.

Supplement: Supplementary file 7 — Additional file 7: Table S3. List of salivary proteins quantified by SWATH MS including signal peak area, fold change (FC) for female versus male saliva, logFC and P-values. [file 13071_2021_5118_MOESM7_ESM.pptx]
