# Supplementary material for: A proteomics informed by transcriptomics insight into the proteome of Ornithodoros erraticus adult tick saliva
Source: Parasit Vectors. 2022 Jan 3;15:1. doi: 10.1186/s13071-021-05118-1 (PMC8722417; doi:10.1186/s13071-021-05118-1)
Supplement: Supplementary file 8 — Additional file 8: Figure S2. Heat map showing levels of differentially expressed proteins (P < 0.05) among female and male biological replicated samples, and hierarchical clustering showing two main clusters comprising F1–F3 samples and M1–M3 samples, corresponding to female and male saliva, respectively. [file 13071_2021_5118_MOESM8_ESM.pptx]

## Slide 1
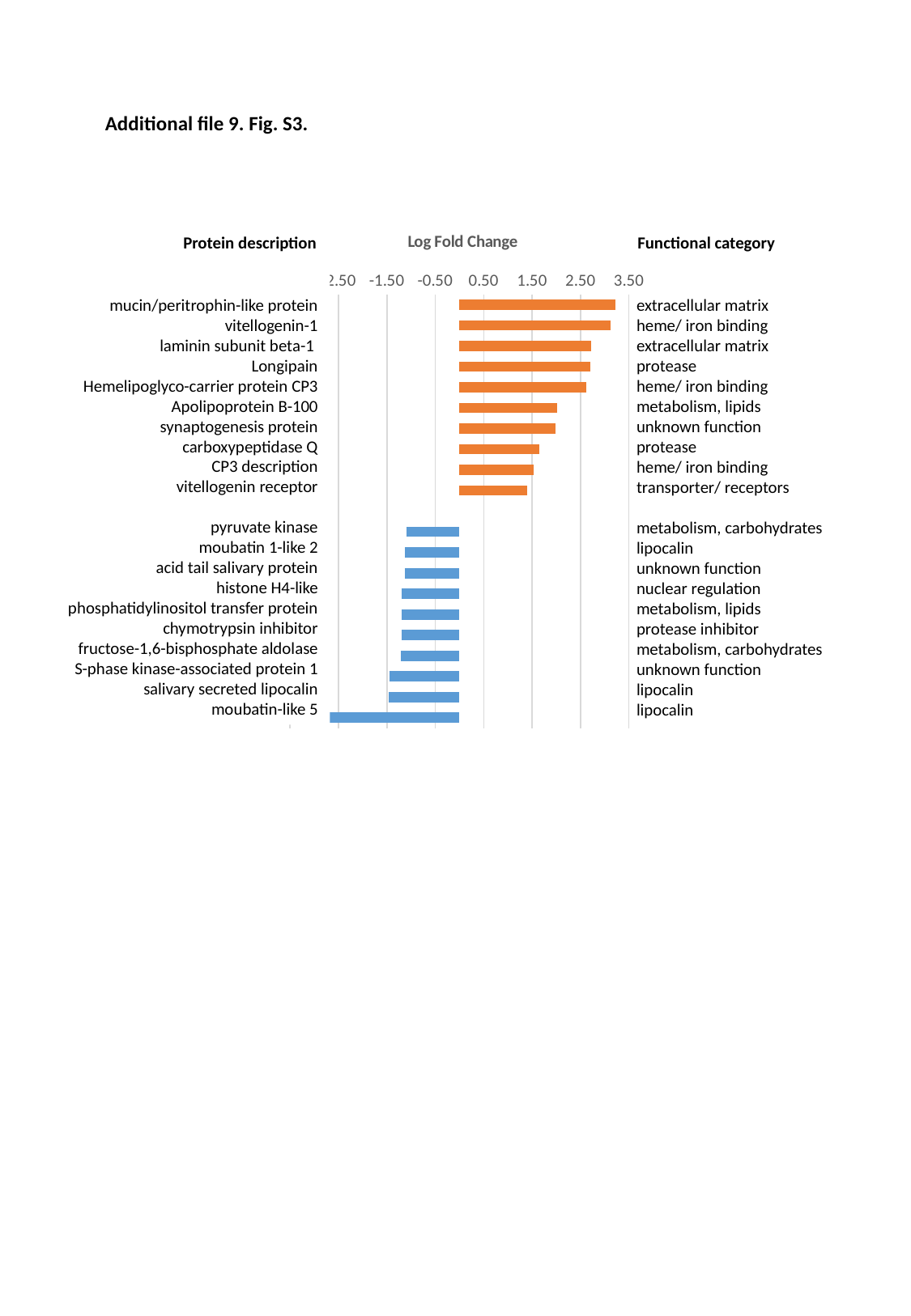

Additional file 9. Fig. S3.
### Chart:
| Category | Log Fold Change |
|---|---|
| mucin/peritrophin-like protein precursor | 3.215954342743633 |
| vitellogenin-1 | 3.124894495392382 |
| laminin subunit beta-1 isoform X1 | 2.7197259526003976 |
| Longipain, putative | 2.699833020130792 |
| hemelipoglyco-carrier protein CP3 | 2.621189940921154 |
| Apolipoprotein B-100, partial | 2.0168634118784685 |
| synaptogenesis protein syg-2-like | 1.9896826047090568 |
| carboxypeptidase Q | 1.6570950089821823 |
| CP3 description | 1.5256240030961314 |
| vitellogenin receptor | 1.4042501367690414 |
| | None |
| pyruvate kinase, putative | -1.1024290454722891 |
| moubatin 1-like 2 | -1.1264624210113203 |
| acid tail salivary protein | -1.1308330885020854 |
| histone H4-like, partial | -1.1893745053105338 |
| phosphatidylinositol transfer protein | -1.1966511187322422 |
| chymotrypsin inhibitor precursor | -1.1972706240457418 |
| fructose-1,6-bisphosphate aldolase | -1.2144183849835735 |
| S-phase kinase-associated protein 1 | -1.4452829043798083 |
| salivary secreted lipocalin | -1.459912980033005 |
| moubatin-like 5 | -2.6882929413928376 |Protein description
Functional category
mucin/peritrophin-like protein vitellogenin-1
laminin subunit beta-1
Longipain
Hemelipoglyco-carrier protein CP3
Apolipoprotein B-100
synaptogenesis protein
carboxypeptidase Q
CP3 description
vitellogenin receptor
pyruvate kinase
moubatin 1-like 2
acid tail salivary protein
histone H4-like
phosphatidylinositol transfer protein
chymotrypsin inhibitor
fructose-1,6-bisphosphate aldolase
S-phase kinase-associated protein 1
salivary secreted lipocalin
moubatin-like 5
extracellular matrix
heme/ iron binding
extracellular matrix
protease
heme/ iron binding
metabolism, lipids
unknown function
protease
heme/ iron binding
transporter/ receptors
metabolism, carbohydrates
lipocalin
unknown function
nuclear regulation
metabolism, lipids
protease inhibitor
metabolism, carbohydrates
unknown function
lipocalin
lipocalin
